# The Complete Mitochondrial Genome of *Platysternon megacephalum peguense* and Molecular Phylogenetic Analysis

**DOI:** 10.3390/genes10070487

**Published:** 2019-06-27

**Authors:** Hongdi Luo, Haijun Li, An Huang, Qingyong Ni, Yongfang Yao, Huailiang Xu, Bo Zeng, Ying Li, Zhimin Wei, Guohua Yu, Mingwang Zhang

**Affiliations:** 1 College of Animal Sciences and Technology, Sichuan Agricultural University, Chengdu 611130, Sichuan, China; 2 Farm Animal Genetic Resources Exploration and Innovation Key Laboratory of Sichuan Province, Sichuan Agricultural University, Chengdu 611130, Sichuan, China; 3 College of Life Science, Sichuan Agricultural University, Ya’an 625014, Sichuan, China; 4 Institute of Millet Crops, Hebei Academy of Agriculture and Forestry Sciences, Shijiazhuang 050051, Hebei, China; 5 Key Laboratory of Ecology of Rare and Endangered Species and Environmental Protection (Guangxi Normal University), Ministry of Education, Guilin 541004, Guangxi, China; 6 Guangxi Key Laboratory of Rare and Endangered Animal Ecology, College of Life Science, Guangxi Normal University, Guilin 541004, Guangxi, China

**Keywords:** mitogenome, gene rearrangement, phylogenetic analysis, divergence time

## Abstract

*Platysternon megacephalum* is the only living representative species of Platysternidae and only three subspecies remain: *P. m. megalorcephalum*, *P. m. shiui*, and *P. m. peguense*. However, previous reports implied that *P*. *m*. *peguense* has distinct morphological and molecular features. The characterization of the mitogenome has been accepted as an efficient means of phylogenetic and evolutionary analysis. Hence, this study first determined the complete mitogenome of *P. m. peguense* with the aim to identify the structure and variability of the *P. m. peguense* mitogenome through comparative analysis. Furthermore, the phylogenetic relationship of the three subspecies was tested. Based on different tRNA gene loss and degeneration of these three subspecies, their rearrangement pathways have been inferred. Phylogenetic analysis showed that *P. m. peguense* is a sister group to (*P. m. megalorcephalum* and *P. m. shiui*). Furthermore, the divergence time estimation of these three subspecies coincided with the uplift of the Tibetan Plateau. This study shows that the genetic distances between *P*. *m*. *peguense* and the other two subspecies are comparable to interspecific genetic distances, for example within *Mauremys*. In general, this study provides new and meaningful insights into the evolution of the three Platysternidae subspecies.

## 1. Introduction

Most vertebrate mitochondrial genomes consist of 13 protein-coding genes (PCGs), 22 transfer RNA (tRNA) genes, two ribosomal RNA (rRNA) genes, and a control region (CR). These control duplication and transcription and range in size from 15 to 20 kb [1,2]. Due to their quick rate of evolution, high copy number, and typical maternal inheritance, the mitochondrial sequences are vital genetic markers for the study of phylogenetic relationships and for species diagnosis, which complements new developments using nuclear genes [3,4,5,6]. To date, the mitogenomes of most turtles follow a typical vertebrate arrangement with the exception of *Malacochersus tornieri* and *Platysternon megacephalum* (the big-head turtle) [7]. However, first report of the *P. megacephalum* mitogenome, which observed large-scale gene rearrangement and duplication of mitochondrial genes (which does not occur in closely related turtles), challenged the viewpoint of mitochondrial stabilization selection in turtles.

*P. megacephalum* is the only living representative species of the turtle lineage of Platysternidae and only three subspecies remain: *P. m. megalorcephalum*, *P. m. shiui*, and *P. m. peguense*, which are mainly distributed throughout China, Vietnam, and Myanmar. However, due to overexploitation and habitat destruction, the wild population has sharply declined [8] and the species have therefore been listed in the IUCN Red List of endangered species (EN) during the early 21th century.

In a previous study, a sister relationship was confirmed between the monotypic *Platysternon* and Emydidae [9]. Although the phylogenetic status of *Platysternon* was definite in Testudines, research about the only remaining three subspecies remains insufficient. An observational study indicated that the morphological data of *P. m. peguense* was particularly distinct compared to the other two subspecies [8]. Furthermore, Zheng et al. [10] reported that among the double CRs of these three subspecies, the CRs of *P. m. peguense* had a unique repeat unit. The phylogenetic tree constructed with these CRs showed that *P. m. megalorcephalum* and *P. m. shiui* were phylogenetically closer than *P. m. peguense*. These reports imply that *P*. *m*. *peguense* has more distinct morphological and molecular features than the other two subspecies. However, the morphological characteristics constantly changed with the development of the individual, which posed limitations [11]. Data on rapidly mutated CRs of mitochondrial fragments are not comprehensive for the understanding of the information of these three subspecies [12].

To further identify and protect these endangered turtles, this study determined the complete mitogenome of *P. m. peguense* by next-generation sequencing (NGS) and Sanger sequencing, and described its characterization and gene rearrangement. Moreover, this study specifically focused on the comparison between the mitogenome of these three subspecies to explore the features that differ between *P. m. peguense* and the other two subspecies. For this, maximum likelihood (ML) and Bayesian inference (BI) methods were used to perform the phylogenetic analyses to provide further information on the only remaining three subspecies of *Platysternon*. Moreover, a divergence time estimation is presented for these species, and the genetic distances between the three subspecies were evaluated. This provides a foundation for the future protection of these species and helps to further understand the phylogeny and evolutionary biology of these endangered turtles.

## 2. Materials and Methods

### 2.1. Ethics Statement, Taxon Sampling, and DNA Extraction

This study on *P. m. peguense* was approved by the Committee of the Ethics on Animal Care and Experiments at Sichuan Agricultural University (CEACE S20174231) and was conducted in accordance with the guidelines stated by the CEACE.

A sample of *P. m. peguense* was collected in Yunnan Province, China in 2016 and muscle tissue of the tail tip was immediately preserved in 99% ethanol. Genomic DNA was extracted from this muscle tissue according to protocols of the Ezup Column Bacteria Genomic DNA Purification Kit (Sangon Biotech, China). The extract was prepared for both Sanger sequencing and NGS.

### 2.2. PCR Amplification and Sequencing

DNA samples were sent to Paisenuo Biotechnology (Shanghai, China) for library construction and sequencing on an Illumina MiSeq platform with 400 bp paired-end reads (PE400). Raw data were cleaned up and filtered to obtain high quality (HQ) data, which were then selected for de novo assemblies by A5-miseq v20150522 [13] and SPAdes v3.9.0 [14] software. The contigs of the mitogenomic sequences were identified by the NT library on NCBI using BLASTn (BLAST C v2.2.31+) and were adjusted by pilon v1.18 to evaluate both accuracy and completeness.

**Figure 1:**
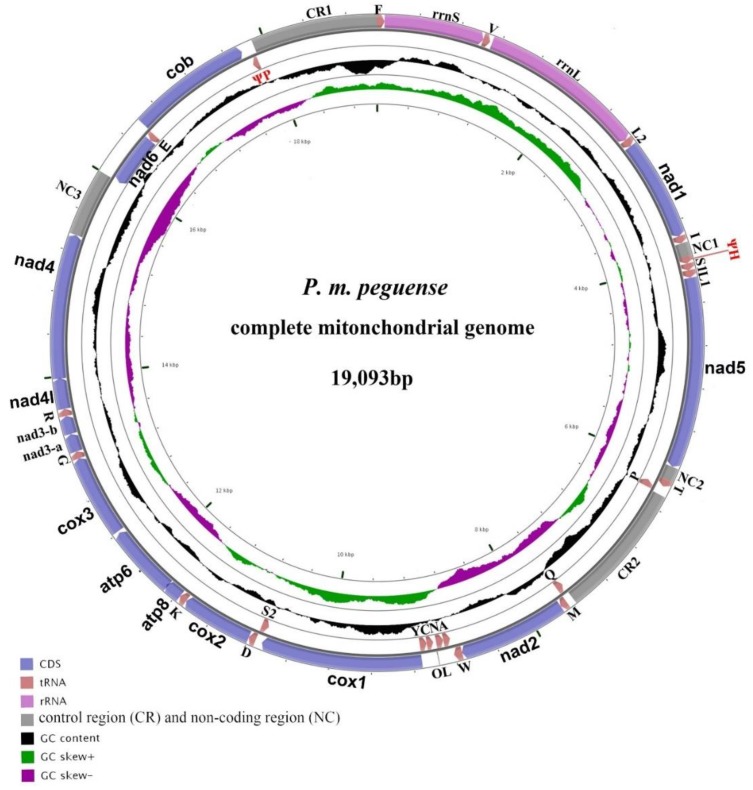
Gene localization in *P. m. peguense*. The direction of gene transcription is indicated by arrows. Blue arrows represent protein-coding genes (PCGs), pink arrows represent rRNA genes, crimson arrows represent tRNA genes and are designated by single-letter amino acid codes, and red font represents degenerate genes. Non-coding region (NC) and control region (CR) are indicated as gray rectangles. OL represents the replication origin of the L strand. The GC content is plotted using a black sliding window and the GC-skew is plotted using green and violet color sliding windows as the deviation from the average in the complete mitogenome. The figure was drawn using CGView [15] with default parameters.

Since highly repetitive sequences were present in the control region, the NGS results showed that the control region 1 (CR1) was missing (Figure 1). Two pairs of primers were designed from known sequences of *P*. *megacephalum* in GenBank (accession numbers KC476449-KC476496) [10] to amplify this region. The following primers were used (listed 5’ to 3’); (A) f1: GGCCAAATAGCCTCCATCCT, r1: AGGTTTAAAATTA CTGTCGCGGA and (B) f2: AACCACATCATTCCGACCAC, r2: CGGCTCCTTTG TCTAATAG. PCR cycling was performed as follows; 94 °C for 5 min for pre-denaturation; 35 cycles of denaturing at 94 °C for 1 min, annealing at 55 °C for 1 min, extension at 72 °C for 2 min 30 s, and a final extension step of 72 °C for 10 min. The PCR products were preliminarily confirmed on a 1.0% agarose gel. The purified nested PCR products were cloned into a pEASY-T5 vector (TransGen, Beijing, China) and transformed into the *Escherichia coli* strain DH5α. Plasmids isolated from single clones were sequenced in both directions to ensure fidelity of each sequence using the ABI 3730XL automated sequencer.

### 2.3. Sequence Assembly, Annotation, and Analysis

Sequence data included data from NGS and Sanger sequencing and the CR1 was performed on the reference sequences of *P. m. peguense* (KC476468-KC476472) [10]. These data were assembled using both MEGA 7 [16] and Dnastar 6.0 software. PCG, rRNA, and tRNA genes were annotated by MITOS web server [17] and tRNAscan-SE [18] to obtain a comparison reference. The graphical map of the mitochondrial genome was drawn by CGview [15]. The relative synonymous codon usage (RSCU), start/stop codon, codon usages, and composition of nucleotides were analyzed in MEGA 7. The skew compositions were calculated using the following equations; AT-skew = (A − T)/(A + T) and GC-skew = (G − C)/(G + C) [19]. In addition, the same methods were used to reanalyze the published mitogenomes of both *P. m. shiui* (DQ256377) and *P. m. megalorcephalum* (DQ016387) to supplement previously unpublished data. Additionally, to explore the level of divergence between *Platysternon* subspecies, they and the 14 different species within four genera of turtles (seven for *Mauremys*, four for *Cuora*, and three for *Testudo*; Table S1) in this study were used to calculate the genetic distances as a comparison between intraspecific and interspecific genetic distances. The genetic distances between each pair of the 13 PCGs of these intraspecific and interspecific comparisons of 17 species were calculated with MEGA 7, using the Kimura-2-parameter (K2P) model.

### 2.4. Phylogenetic Analysis and Divergence Time Estimation

To determine the phylogenetic position of *P. m. peguense*, 34 turtles (including *P. m. peguense*) representing 12 Testudines families and five outgroups species (Table S1) were included for this analysis [20]. The complete sequences of 13 PCGs of all species with deletions in the stop codons were aligned through MAFFT using default parameters [21], and MEGA 7 was used to translate nucleotide to amino acid sequences. 

The concatenated set of nucleotide and amino acid sequences was used for phylogenetic analysis, which was performed with the ML and Bayesian inference (BI) methods using raxmlGUI [22] and Mrbayes v.3.2.2 [23], respectively. PartitionFinder 2.1.1 was used to select the nucleotide and amino acid substitution model of 13PCGs for the construction of ML and BI phylogenetic trees, using two runs for raxmlGUI and MrBayes both for the nucleotide and the amino acid sequences, respectively. For nucleotide sequences, 13 PCGs were codon-partitioned (Table S2). For the amino acid sequences, the best-fit model of each partition was selected (Table S2). All search models for the 13 PCGs of sequences were set to the “raxml” and/or “mrbayes” runs command line option under the greedy algorithm using “linked” branch lengths [24]. All the best partitioning schemes for ML and BI analyses are show in the Supplementary Table S2. For ML analysis, 1000 bootstrap replicates were used to calculate the bootstrap of the program. In the BI analysis, two independent runs for 10^7^ Markov chain Monte Carlo (MCMC) generations with four chains and sampling trees occurred every 1000 generations. The first 25% of trees were discarded as burn-in samples and the remaining trees were used to generate Bayesian consensus trees. FigTree 1.4.2 was used to visualize and edit the results of both raxmlGUI and Bayesian trees.

BEAST v2.5.2 [25] was used to estimate the molecular timing analyses based on the 39 species dataset. Four time constraints were imposed to calibrate the molecular clock: (1) the split between *Carassius auratus* and *Tylototriton verrucosus* was constrained to be 413–443 Mya based on fossilized *C. auratus* [26]; (2) the split between *Crocodylus porosus* and *Alligator sinensis* was constrained to be 77–89 Mya based on the fossilized *C. porosus* [27]; (3) the most recent common ancestor (MRCA) of *Gallus gallus* and *Crocodylus porosus* was estimated to be 229–244 Mya [28]; and (4) the MRCA of both Cryptodira and Pleurodira was estimated to be 181–208 Mya [29]. Molecular dating involved a Yule speciation tree and normally distributed priors. TRACER 1.7 was used to confirm whether the output reached stationarity [30], and a relaxed molecular clock with uncorrelated lognormal distribution was specified. Four independent chains of 10^7^ samples were run for the Markov chain Monte Carlo (MCMC) analysis, and the first 25% of all samples were discarded as burn-in using TreeAnnotator.

## 3. Results and Discussion

### 3.1. Sequencing Data and Genome Composition

The assembled mitogenome of *P. m. peguense* was 19,093 bp in length, including 13 PCGS, 23 tRNA genes (including an additional copy of *trnP* (Ψ)), two rRNA genes, two control regions, and three non-coding spaces (Figure 1). The OL is located in the tRNA cluster “*WANCY*”. Nine tRNA genes and *nad6* were encoded by the L-strand (−), and the remaining genes were encoded by the H-strand (+) (Figure 1 & Table 1). Overall, the nucleotides of *P. m. peguense* mitogenome sequences were A = 33.8%, T = 27.4%, C = 25.9%, and G = 12.9%. 

Table 2 shows that in *Platysternon*, PCGs had the lowest A+T content (from 59.7% to 59.9%), while CRs has the highest (from 66.3% to 68.1%). Among the three subspecies, the A+T contents were similar, which indicates that the base composition of the sequence was extremely conservative. However, the GC-skew values in tRNA genes of *P. m. shiui* were negative (−0.053) and the AT-skew values in CRs of *P. m. peguense* were positive (0.011), which differs from the other two subspecies. This indicates that in *P. m. shiui*, more Cs than Gs occur in tRNA genes, and a bias exists toward Ts in *P. m. peguense* of CRs. In general, the complete mitogenome indicates that the base composition of the three subspecies is very similar even if a difference in the proportion of bases in specific regions exists.

### 3.2. PCGs and Codon Usage

The mitogenome of *P. m. peguense* PCGs has a length of 11,388 bp. Despite *nad6* being encoded by the L-strand (−), the remainder of the genes (*nad1*, *nad2*, *nad3*, *nad4l*, *nad4*, *cox1*, *cox2*, *cox3*, *atp8*, *atp6*, and *cob*), including the translocation *nad5*, are all encoded by the H-strand (+). The stop codon of 10 PCGs is TAA or TAG, and includes incomplete stop codons (T or TA), while *cox1* and *nad6* genes use AGG as stop codon (Table 1). The mitogenome of the three subspecies of *Platysternon* showed that all PCGs shares the same start and stop codons, except for *P*. *m*. *peguense*, which has a specific start codon in *cox1* (GTG instead of ATG) and *atp6* (ATG instead of ATA). Stop codons also differ in *cox2* (TAG instead of TAA) and *nad6* (AGG instead of AGA) and only *P*. *m*. *peguense* has a complete stop codon TAA in *cob* (Table S3).

**Figure 2:**
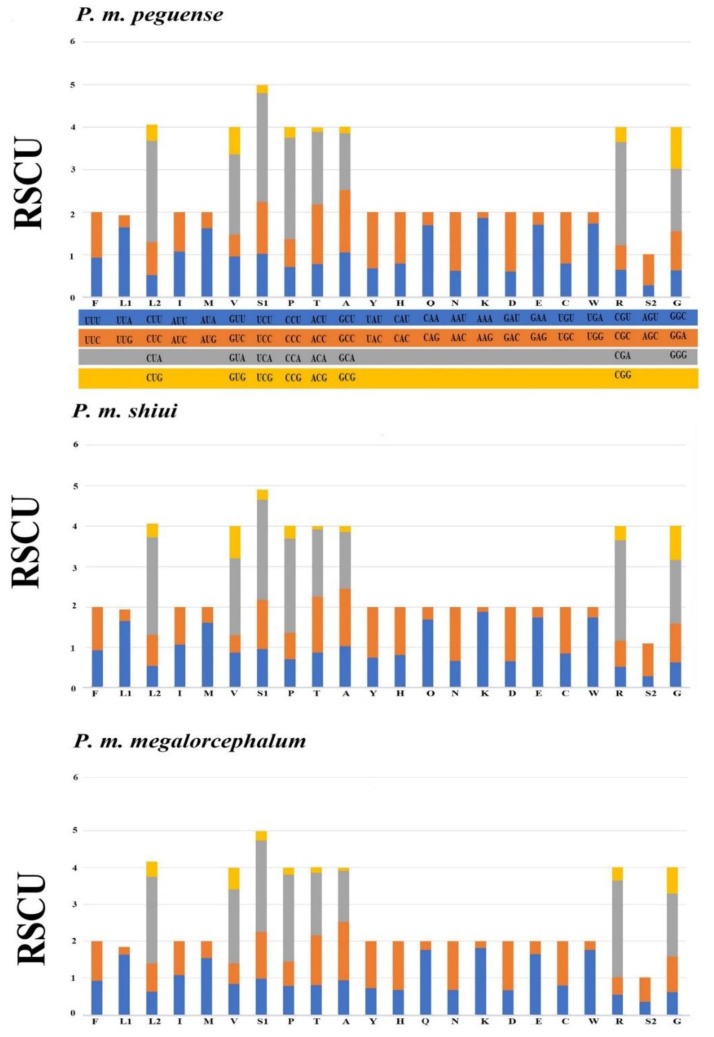
Relative synonymous codon usage (RSCU) in the mitogenomes of three subspecies of *Platysternon*. The codon-encoded amino acids are labeled according to the IUPAC-IUB single-letter amino acid coding, which excludes both the start codon and the stop codon. The different colors in the column chart represent the codon families corresponding to below amino acids, and the three subspecies use consistent colors to represent the same codon families.

The Relative Synonymous Codon Usage (RSCU) indicates that six codon families (UUA, CUA, AUU, AUA, AUC, and ACA) are most commonly used in *P. m. peguense*, with more than 130 instances (Table S4). A comparison of RSCU of the three subspecies showed that, with the exception that *P. m. peguense* did not use the stop codon AGA, codon usage was very consistent (Figure 2). This result shows that the most common amino acids are Leu, Val, Ser, Thr, Ala, Arg, and Gly in these three subspecies.

### 3.3. Transfer RNAs and Control Region

**Figure 3:**
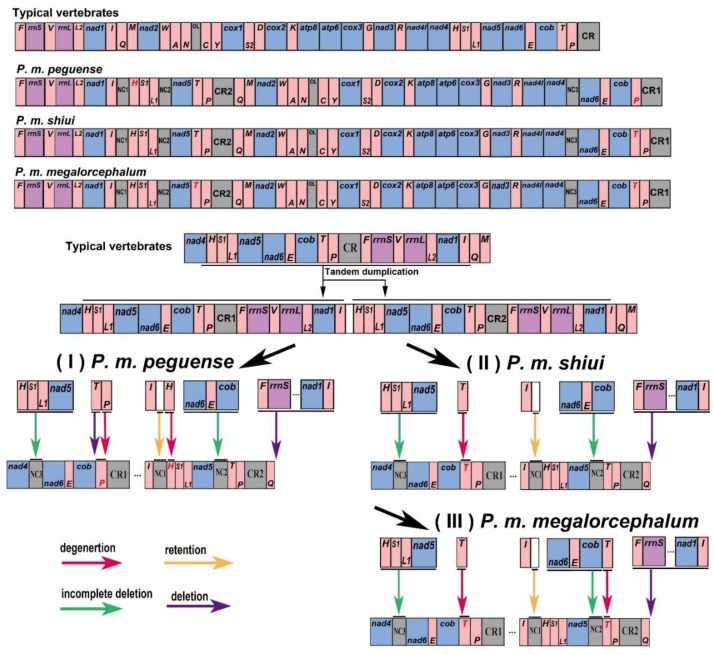
Mitogenome features and putative gene rearrangement process of *P. m. peguense* (type (I)), *P. m. shiui* (type (II)), and *P. m. megalorcephalum* (type III) of the three investigated *Platysternon* subspecies. The gene name above the median indicates encoding by the H-strand (+), while the gene name below the median indicates encoding by the L-strand (−). Red font indicates degenerated tRNA genes, and tRNA genes are designated using single-letter amino acid codes.

**Figure 4:**
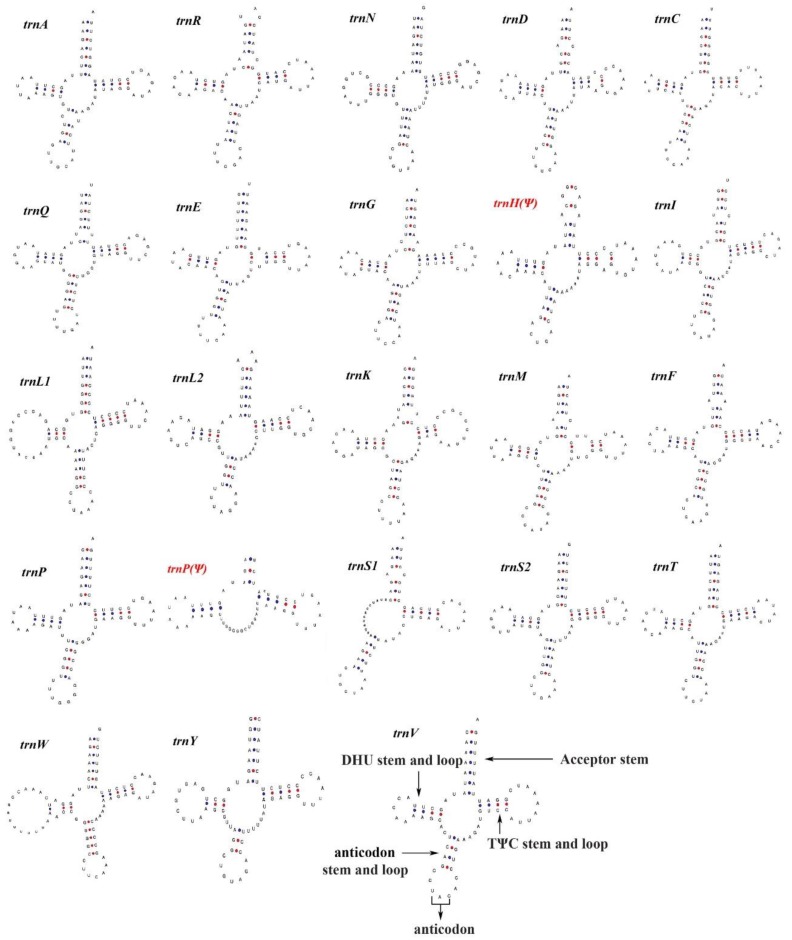
Putative secondary structures for 23 tRNA genes of *P. m. peguense*. The tRNA genes are labeled with standard abbreviations. Red font indicates degenerated tRNA genes.

A total of 23 tRNA genes can be found in the mitogenome of *P. m. peguense*, including an additional copy of *trnP* (Ψ); however, the *trnT* (Ψ) at CR1 5’ is missing compared to the other two subspecies (Figure 3). The tRNA genes range in size from 66 to 79 bp. Among them, nine tRNA genes are located on the L-strand (−) and the remaining 14 are located on the H-strand (+) (Table 1). Most of the tRNA genes fold into the typical clover-leaf structure (Figure 4). Even a lack of the dihydrouridine (DHU) arm in *trnS1* does not affect its function [31,32,33,34], which has been reported in many insects [35]. However, despite the potential of *trnP* (Ψ) to fold into a secondary structure, it lacks anticodon stems and loop, while the anticodon sequences of *trnH* (Ψ) degenerated from GUG to CUG, indicating a loss of tRNA function. Previous studies indicated that the missing function of the tRNA genes may have imported nuclear copies as supplement [36,37].

Most metazoan mitogenomes contain only one control region; however, more recent studies have reported multiple CRs in the mitogenome of specific animals [38]. Like the other two previously reported subspecies [10], *P. m. peguense* generates two CRs, which are located between *trnF* and *trnP* (Ψ) (CR1) and between *trnP* and *trnQ* (CR2) (Figure 1). These CRs have the highest A+T content (66.3%) in the mitogenome of *P. m. peguense* (Table 2). However, the AT-skew is positive, which is unique in *P. m. peguense* (Table 2), indicating more As than Ts, which caused by the unique A-T long tandem repeats in the CRs of *P. m. peguense* [10]. Three important functional sections (TAS, CD, and CSB) in the CRs were present in all three subspecies [10], indicating that neither of the two CRs in *P. m. peguense* had degenerated. This indicates natural mitogenome selection toward high-speed replication needs, since it has been reported that a double CR mitogenome can replicate more efficiently [39].

### 3.4. Gene Rearrangement

In contrast to other turtle species, the mitogenome of *Platysternon* features a large-scale gene rearrangement and duplication of CRs (Figure 3). For *P. m. peguense*, as reported previously for both others subspecies, a highly similar fragment of CR1 that CR2 has been inserted between the *trnI* and *trnP*. The gene cluster (*trnH*/*trnS1*/*trnL1*/*nad5*) was translocated [40]. However, compared to the gene arrangements of the three subspecies of *Platysternon*, *P. m*. *peguense* lost the *trnT* (Ψ) at the 5’ end of CR1, and the *trnP* (Ψ) and *trnH* (Ψ) degenerated into a pseudogene. Regarding the different gene rearrangements for the three subspecies, their rearrangement pathways were re-inferred under the TDRL model (Figure 3). 

The first copy of the *trnH*-*nad5* region and the second copy of the *nad6*-*cob* region were incompletely deleted and generated NC3 and NC2, respectively. In addition, the retention of the partial duplication of *nad4* led to NC1, and these processes are identical between type (I) (*P. m. peguense*), type (II) (*P. m. shiui*), and type (III) (*P. m. megalorcephalum*) (Figure 3). In type (I), the *trnT* near the CR1 was deleted, and *trnP* (Ψ) and *trnH* (Ψ) degenerated to pseudogenes. In contrast to type (I), in type (II), only the *trnT* (Ψ) near *cob* degenerated. In the type (III), both *trnT* (Ψ) degenerated. This suggests that the common ancestor of these three subspecies underwent different mitochondrial genetic recombination events and genetic processes were inherited and retained, resulting in differentiation into the three subspecies. Additionally, in three subspecies of *Platysternon*, *nad3* contains an additional cytosine (C) base at position 174. It has had been reported for many turtles that this additional nucleotide causes a translational frameshift in *nad3* [41,42,43]. However, in *P. m. megalorcephalum*, *nad1* contains three additional bases at positions 253, 524, and 612 [6], which are not found in *P. m. peguense* and *P. m. shiui*.

### 3.5. Phylogenetic Analysis

**Figure 5:**
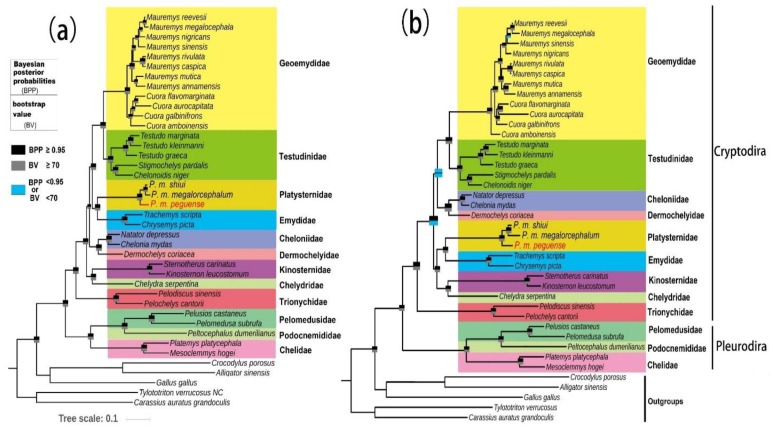
Phylogenetic tree of *Platysternon* and turtles based on the concatenated (**a**) nucleotide and (**b**) amino acid sequences of 13 PCGs both using maximum likelihood (ML) and Bayesian inference (BI) analysis. At each node, the upper rectangle indicates the Bayesian posterior probability (BPP), used for the Mrbayes analyses, while the lower rectangle bootstrap value (BV) was used for the RaxML analyses. Species specific taxonomic ranks were incorporated with specific clades.

Phylogenetic analysis was performed on the nucleotide (11,439 bp) and amino acid (3813 amino acid positions) sequences of 13 PCGs, derived from 34 complete mitogenomes of Testudines from 12 families, and five species were selected as outgroups. The nucleotide and amino acid tree topology structures obtained via BI and ML analyses, respectively, were consistent (Figure 5). The main differences between the nucleotide and amino acid trees were the phylogenetic placements of (Cheloniidae + Dermochelyidae) and (Chelydridae + Kinosternidae). Moreover, a number of support values of their related nodes were also different.

In the present analyses of the *Platysternon* lineage, *P. m. peguense* was sister group to *P. m. megalorcephalum* and *P. m. shiui* with strong support (BPP ≥ 0.95; BV ≥ 70) both in nucleotide and amino acid trees (Figure 5). This was confirmed at the mitogenome level—a relationship that has been suggested before via analysis of only CRs data [10]. Within Cryptodira, this study confirmed the placement of *Platysternon* as sister to Emydidae and formed a group distinct from the rest of the families both in nucleotide and amino acid trees (BPP ≥ 0.95; BV ≥70), as indicated by analysis of previous studies [9,40,44,45]. Crawford et al. [9] suggested to phylogenetically define the name ‘Emysternia’ in reference to the clade originating from the most recent common ancestor of *Platysternon* and Emydidae. In contrast, the nucleotide tree of this study (Figure 5a) showed that Chelydridae and Kinosternidae form a monophyletic group, which was well supported (BPP ≥ 0.95; BV ≥ 70) (Figure 5a). This differed from the results of Crawford et al. [9] and Pereira et al. [45], which, based on nuclear and mitochondrial data, indicated this was a sister relationship of ((Chelydridae + Kinosternidae) + (Cheloniidae + Dermochelyidae)). In this context, the placements of (Cheloniidae + Dermochelyidae) and (Chelydridae + Kinosternidae) are most likely due to anomalies in the evolution of the molecules, such as uneven rates of substitution and/or long branch attraction [46,47,48]. However, the amino acid tree (Figure 5b) indicated a sister group of ((Chelydridae + Kinosternidae) + (Emydidae + *Platysternon*)) with good support (BPP ≥ 95; BV ≥ 70), but with low support for both the sister group of ((Cheloniidae + Dermochelyidae) + (Testudinidae + Geoemydidae)) (BPP < 0.95; BV < 70) and between the two major clades ((Chelydridae + Kinosternidae + Emydidae + *Platysternon*) + (Cheloniidae + Dermochelyidae + Testudinidae + Geoemydidae)) (BPP ≥ 95; BV < 70) (Figure 5b). These results are retrieved only in the amino acid tree in this study. This provides new insight on the phylogeny of the species, although several the nodes of amino acid tree had low support values. In summary, more taxon sampling and more sequence data, including mitochondrial and nuclear markers, are required to obtain a more robust phylogenetic framework.

### 3.6. Divergence Time Estimation

**Figure 6:**
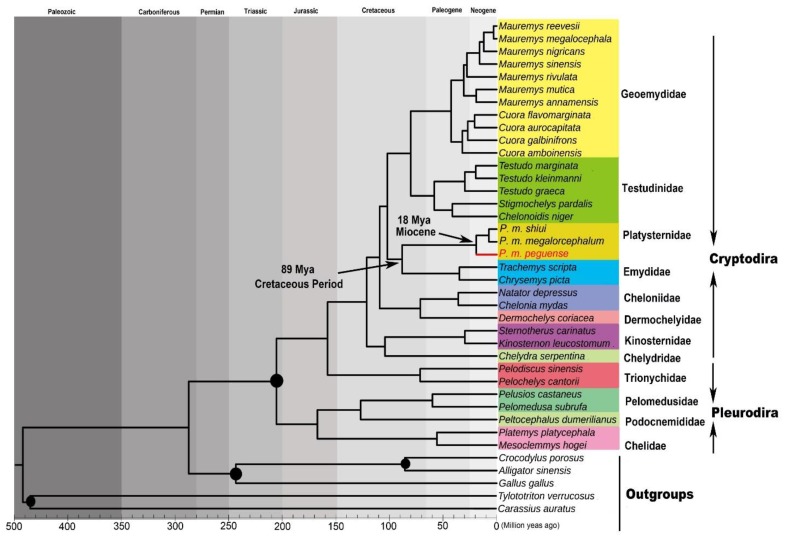
Time tree of *Platysternon* and turtles based on the concatenated nucleotide sequences of 13 PCGs using BEAST analysis. Four nodes were used for fossil-calibrated time points (solid black circles). Species specific taxonomic ranks were incorporated with specific clades.

Based on the time trees of turtles in this study (Figure 6), the origin of (Emydidae + *Platysternon*) can be placed during the late Cretaceous Period (~89 Mya), which is consistent with the estimation of Lourenço et al [49]. During this time period, the trend toward continental fission and marine ingression reached its climax [50], and oceanic anoxic events lowered the bottom water oxygen concentrations, leading to significant biological extinction and forced migration [51]. These reasons may have led to the change from marine life to offshore or terrestrial life, and genetic differentiation occurred due to the increased complexity of living environments. The MRCA of the three subspecies of *Platysternon* was estimated at the Miocene (~18 Mya) (Figure 6), during which the Tibetan Plateau was fully uplifted [52]. This important tectonic event substantially changed local landmasses and river distributions, thus causing the rapid extinction or differentiation of many species [53,54,55]. On the edge of the Tibetan Plateau, this uplifting altered the terrain of habitats from gentle to steep, which formed a barrier for species distribution and resulted in species divergence and speciation of *Platysternon* due to their weak migration ability. The uplifted habitat divided the distribution of *Platysternon* in China and Myanmar, and limited the genetic exchange of *P. m. peguense*, and even more frequently between *P. m. shiui* and *P. m. megalorcephalum*. Furthermore, considering the divergence time estimation of the three subspecies and the influence of geographical factors, the fact that *P. m. shiui* has a closer phylogenetic relationship to *P. m. megalorcephalum* and the mitochondrial genetic differences between *P. m. peguense* and the other two subspecies may be a response to the occurred uplifting. This further confirmed the occurrence of the different mitochondrial genetic recombination events found in this study. 

### 3.7. Genetic Divergence

In the analysis of genetic distances based on 17 species within four genera, the results of pairwise genetic distances of 13 PCGs showed that *P*. *m*. *megalorcephalum* and *P*. *m*. *shiui* had minimum genetic distances (0.024) compared to other turtles (Table 3). This further confirmed their close relationship and low differentiation. Significantly, the genetic distances between *P*. *m*. *peguense* and *P*. *m*. *megalorcephalum* (0.068) and between *P*. *m*. *shiui* (0.059) were well within the range of interspecific distances of *Mauremys* (0.040–0.094). However, they did not reach the interspecies genetic distances within *Testudo* (0.71–0.89) and *Cuora* (0.83–0.116). These results confirm that *P*. *m*. *peguense* is more highly differentiated than the other two subspecies. Although this has previously been suggested, the complete 13 PCGs comparison can obtain more genetic information than single gene fragments, and thus reduces the effects of specific mitochondrial gene fragments [56]. The analysis presented, in combination with the species of 13 PCGs, showed that the genetic distances between *P*. *m*. *peguense* and other two subspecies was comparable to interspecies genetic distances within the *Mauremys*; however, *P*. *m*. *megalorcephalum* and *P*. *m*. *shiui* had the lowest genetic distances among the tested turtles. These results suggest that *P*. *m*. *peguense* likely reached the level of differentiation comparable to species of *Platysternon* and may have formed a new species. On the other hand, the genetic difference may have gradually accumulated with geographical isolation, leading to further genetic differentiation between *P*. *m*. *peguense* and the other two subspecies. In summary, the reconstructed phylogenetic tree, the divergence time estimation, and pairwise genetic distances support this view.

## 4. Conclusions

In conclusion, this study determined the complete mitogenome of *P. m. peguense* via NGS and Sanger sequencing, characterized the mitogenome, provided phylogenetic analyses of *P. m. peguense* and confirmed that *P. m. peguense* has greater genetic differentiation than the other two investigated subspecies. Furthermore, the divergence time estimation of the three subspecies investigated in this study coincided with the uplifting of the Tibetan Plateau, suggesting that geology may have influenced the speciation of *P*. *m*. *peguense*. Moreover, the presented analysis shows that the genetic distances between *P*. *m*. *peguense* and the other two subspecies is well within the range of interspecific distances of *Mauremys*. With the disclosure of different gene arrangements, phylogenetic placements, and genetic distances in the three subspecies of *Platysternon*, the taxonomy and evolutionary history of these three subspecies should be reconsidered. Further sampling from different taxonomic grades and both their mitogenome and nuclear data will improve our understanding of the phylogenetic and evolutionary biology of the only remaining three subspecies of *Platysternon*.

## Figures and Tables

**Table 1:** Annotation of the complete mitogenome of *P. m. peguense*.

Gene	Direction	Location	Size (bp)	Anticodon	Start Codon	Stop Codon	Intergenic Nucleotides
*trnF*	+	1–70	70	GAA			0
*rrnS*	+	71–1028	958				−1
*trnV*	+	1028–1096	69	TAC			21
*rrnL*	+	1118–2702	1585				−1
*trnL2*	+	2702–2777	76	TAA			0
*nad1*	+	2778–3749	972		ATG	TAG	−1
*trnI*	+	3749–3819	71	GAT			131
*trnH* (Ψ)	+	3951–4017	67	CTG			0
*trnS1*	+	4018–4083	66	GCT			−1
*trnL1*	+	4083–4154	72	TAG			1
*nad5*	+	4156–5976	1821		ATG	TAA	135
*trnT*	+	6112–6182	71	TGT			1
*trnP*	−	6184–6253	70	TGG			15
CR2		6267–7553	1286				−1
*trnQ*	−	7553–7623	71	TTG			−1
*trnM*	+	7623–7691	69	CAT			0
*nad2*	+	7692–8732	1041		ATG	TAG	−2
*trnW*	+	8731–8809	79	TCA			1
*trnA*	−	8811–8879	69	TGC			1
*trnN*	−	8881–8954	74	GTT			3
*OL*	+	8958–8984	27				−2
*trnC*	−	8983–9048	66	GCA			0
*trnY*	−	9049–9119	71	GTA			1
*cox1*	+	9121–10,668	1548		GTG	AGG	−9
*trnS2*	−	10,660–10,730	71	TGA			3
*trnD*	+	10,734–10,803	70	GTC			0
*cox2*	+	10,804–11,490	687		ATG	TAG	1
*trnK*	+	11,492–11,563	72	TTT			1
*atp8*	+	11,565–11,732	168		ATG	TAA	−16
*atp6*	+	11717–12,399	683		ATG	TA(A)	18
*cox3*	+	12,418–13,202	785		ATG	TA(A)	−1
*trnG*	+	13,202–13,271	70	TCC			0
*nad3-a*	+	13,272–13,445	174		ATG	---	1
*nad3-b*	+	13,447–13,621	175		---	T(AA)	0
*trnR*	+	13,622–13,690	69	TCG			0
*nad4l*	+	13,691–13,987	297		ATG	TAA	−7
*nad4*	+	13,981–15,358	1378		ATG	T(AA)	640
*nad6*	−	15,999–16,523	525		ATG	AGG	0
*trnE*	−	16,524–16,591	68	TTC			4
*cob*	+	16,596–17,762	1167		ATG	TAA	48
*trnP* (Ψ)	−	17,811–17,872	62	---			17
CR1		17,890–19,093	1203				0

**Table 2:** Base composition and skewness of the *Platysternon* mitogenomes of three subspecies.

Species	Total Size (bp)	Complete Mitogenome			tRNAs			PCGs			rRNAs			CRs		
		A + T%	AT-skew	GC-skew	A + T%	AT-skew	GC-skew	A + T%	AT-skew	GC-skew	A + T%	AT-skew	GC-skew	A + T%	AT-skew	GC-skew
*P. m. peguense*	19,093	61.2	0.106	−0.335	61.9	0.040	0.031	59.9	0.034	−0.313	60.2	0.236	−0.149	66.3	0.011	−0.344
*P. m. megalorcephalum*	19,196	61.5	0.106	−0.323	61.9	0.048	0.020	59.9	0.034	−0.313	60.0	0.236	−0.149	68.1	−0.008	−0.223
*P. m. shiui*	19,043	61.4	0.107	−0.329	62.3	0.071	−0.053	59.7	0.033	−0.311	60.3	0.237	−0.147	66.9	−0.006	−0.256

**Table 3:** Genetic distances based on 13 PCGs of turtles from 17 species of four genera.

		1	2	3	4	5	6	7	8	9	10	11	12	13	14	15	16	17
1	***P. m. peguense***																	
2	***P. m. megalorcephalum***	0.068																
3	***P. m. shiui***	0.059	0.024															
4	***M. annamensis***	0.257	0.266	0.255														
5	***M. megalocephala***	0.268	0.271	0.259	0.094													
6	***M. mutica***	0.255	0.260	0.250	0.058	0.086												
7	***M. reevesii***	0.261	0.264	0.253	0.087	0.051	0.080											
8	***M. nigricans***	0.260	0.265	0.253	0.089	0.049	0.081	0.040										
9	***M. rivulata***	0.253	0.261	0.251	0.081	0.082	0.079	0.076	0.074									
10	***M. sinensis***	0.260	0.266	0.256	0.090	0.055	0.081	0.049	0.047	0.077								
11	***T. graeca***	0.245	0.253	0.242	0.198	0.198	0.194	0.194	0.193	0.190	0.195							
12	***T. kleinmanni***	0.251	0.255	0.245	0.201	0.201	0.195	0.197	0.194	0.197	0.198	0.089						
13	***T. marginata***	0.246	0.253	0.244	0.201	0.202	0.195	0.196	0.193	0.193	0.196	0.082	0.071					
14	***C. amboinensis***	0.259	0.266	0.256	0.118	0.121	0.114	0.115	0.114	0.113	0.114	0.194	0.198	0.194				
15	***C. aurocapitata***	0.277	0.286	0.276	0.127	0.123	0.126	0.119	0.122	0.121	0.121	0.218	0.222	0.219	0.116			
16	***C. flavomarginata***	0.260	0.268	0.258	0.110	0.112	0.107	0.107	0.109	0.105	0.111	0.193	0.201	0.196	0.099	0.085		
17	***C. galbinifrons***	0.257	0.262	0.251	0.106	0.110	0.106	0.105	0.105	0.102	0.105	0.192	0.194	0.195	0.092	0.096	0.083	

## Supplementary Materials

The following are available online at https://www.mdpi.com/2073-4425/10/7/487/s1,Table S1: List of the mitogenomes of both turtle and outgroup species, analyzed in this study, and their GenBank accession numbers. The represented species in bold were used to calculate the genetic distances, Table S2: Best partitioning scheme selected by PartitionFinder, Table S3: Usage of start and stop codons in the mitogenomes of three subspecies of *Platysternon*, Table S4: Codon number and RSCU of *P. m. peguense* mitochondrial PCGs.
