# Supplementary material for: The Complete Mitochondrial Genome of Platysternon megacephalum peguense and Molecular Phylogenetic Analysis
Source: Genes (Basel). 2019 Jun 27;10(7):487. doi: 10.3390/genes10070487 (PMC6678547; doi:10.3390/genes10070487)
Supplement: Supplementary file 1 [file genes-10-00487-s001.pdf]

Supplementary Materials

Table S1: List of the mitogenomes of both turtle and outgroup species, analyzed in this study, and their GenBank accession numbers. The represented species in bold were used to calculate the genetic distances.

Family	Genus	Species	GenBank No.
Chelidae	*Platemys*	*Platemys platycephala*	KC692464
Chelidae	*Mesoclemmys*	*Mesoclemmys hogei*	NC_036346
Cheloniidae	*Natator*	*Natator depressus*	JX454975
Cheloniidae	*Chelonia*	*Chelonia mydas*	JX454990
Chelydridae	*Chelydra*	*Chelydra serpentina*	NC_011198
Dermochelyidae	*Dermochelys*	*Dermochelys coriacea*	MF460363
Emydidae	*Chrysemys*	*Chrysemys picta*	NC_002073
Emydidae	*Trachemys*	*Trachemys scripta*	NC_011573
Kinosternidae	*Kinosternon*	*Kinosternon leucostomum*	FJ915117
Kinosternidae	*Sternotherus*	*Sternotherus carinatus*	HQ114563
Pelomedusidae	*Pelusios*	*Pelusios castaneus*	KC692463
Pelomedusidae	*Pelomedusa*	*Pelomedusa subrufa*	NC_001947
Trionychidae	*Pelodiscus*	*Pelodiscus sinensis*	AY962573
Podocnemididae	*Peltocephalus*	*Peltocephalus dumerilianus*	AB970731
Trionychidae	*Pelochelys*	*Pelochelys cantorii*	JN016747
Alligatoridae	*Alligator*	*Alligator sinensis*	NC_004448
Cyprinidae	*Carassius*	*Carassius auratus*	AP011239
Phasianidae	*Gallus*	*Gallus gallus*	AB086102
Salamandridae	*Tylototriton*	*Tylototriton verrucosus*	NC_017871
Crocodylidae	*Crocodylus*	*Crocodylus porosus*	AJ810453
Testudinidae	*Stigmochelys*	*Stigmochelys pardalis*	DQ080041
Testudinidae	*Chelonoidis*	*Chelonoidis niger*	JN999704
**Geoemydidae**	***Mauremys***	***Mauremys sinensis***	**FJ871126**
**Geoemydidae**	***Mauremys***	***Mauremys megalocephala***	**HM132059**
**Geoemydidae**	***Mauremys***	***Mauremys annamensis***	**NC_017875**
**Geoemydidae**	***Mauremys***	***Mauremys reevesii***	**KJ700438**
**Geoemydidae**	***Mauremys***	***Mauremys mutica***	**KP938957**
**Geoemydidae**	***Mauremys***	***Mauremys nigricans***	**KT951839**
**Geoemydidae**	***Mauremys***	***Mauremys rivulata***	**NC_029183**
**Geoemydidae**	***Cuora***	***Cuora galbinifrons***	**EU809939**
**Geoemydidae**	***Cuora***	***Cuora aurocapitata***	**AY874540**
**Geoemydidae**	***Cuora***	***Cuora flavomarginata***	**EU708434**
**Geoemydidae**	***Cuora***	***Cuora amboinensis***	**FJ763736**
**Testudinidae**	***Testudo***	***Testudo kleinmanni***	**DQ080048**
**Testudinidae**	***Testudo***	***Testudo graeca***	**DQ080050**
**Testudinidae**	***Testudo***	***Testudo marginata***	**DQ080047**
**Platysternidae**	***Platysternon***	***P. m. megalorcephalum***	**DQ016387**
**Platysternidae**	***Platysternon***	***P. m. shiui***	**DQ256377**
**Platysternidae**	***Platysternon***	***P. m. megalorcephalum***	**this study**

Table S2: Best partitioning scheme selected by PartitionFinder.

Data matrix	Subset Partitions	Best Model
13PCGS-nucleotide-ML	1: *nad1*_pos1, *cob*_pos1	GTR+I+G
	2: *nad4*_pos2, *nad1*_pos2, *atp6*_pos2	GTR+I+G
	3: *nad3*_pos3, *nad1*_pos3, *nad4l*_pos3, *nad4*_pos3, *atp6*_pos3	GTR+I+G
	4: *nad2*_pos1, *nad5*_pos1	GTR+I+G
	5: *nad2*_pos2, *atp8*_pos2	GTR+G
	6: *nad2*_pos3	GTR+G
	7: *cox1*_pos1, *cox3*_pos1	GTR+I+G
	8: *cox3*_pos2, *cox1*_pos2	GTR+I+G
	9: *cox1*_pos3	GTR+G
	10: *cox2*_pos1	GTR+I+G
	11: *cox2*_pos2	GTR+G
	12: *atp8*_pos3, *cox2*_pos3, *cox3*_pos3	GTR+G
	13: *atp8*_pos1	GTR+G
	14: *atp6*_pos1, *nad4*_pos1	GTR+I+G
	15: *nad3*_pos1, *nad4l*_pos1	GTR+G
	16: *nad4l*_pos2, *nad3*_pos2	GTR +G
	17: *nad5*_pos2	GTR+I+G
	18: *nad5*_pos3	GTR+G
	19: *nad6*_pos1	GTR+G
	20: *nad6*_pos2	GTR+G
	21: *nad6*_pos3	GTR+G
	22: *cob*_pos2	GTR+I+G
	23: *cob*_pos3	GTR+G
13PCGS-nucleotide -BI	1: *nad1*_pos1, *cob*_pos1	GTR+I+G
	2: *nad4*_pos2, *nad1*_pos2, *atp6*_pos2	GTR+I+G
	3: *nad1*_pos3, *nad4l*_pos3, *nad4*_pos3, *atp6*_pos3	GTR+I+G
	4: *nad2*_pos1, *nad5*_pos1	GTR+I+G
	5: *nad2*_pos2, *atp8*_pos2	GTR+G
	6: *nad2*_pos3	GTR+G
	7: *cox1*_pos1, *cox3*_pos1	SYM+I+G
	8: *cox3*_pos2, *cox1*_pos2	GTR+I+G
	9: *cox1*_pos3	GTR+G
	10: *cox2*_pos1	GTR+I+G
	11: *cox2*_pos2	GTR+G
	12: *atp8*_pos3, *cox2*_pos3, *cox3*_pos3	GTR+G
	13: *atp8*_pos1	GTR+G
	14: *atp6*_pos1, *nad4*_pos1	GTR+I+G
	15: *nad3*_pos1, *nad4l*_pos1	GTR+G
	16: *nad4l*_pos2, *nad3*_pos2	GTR+G
	17: *nad3*_pos3	HKY+G
	18: *nad5*_pos2	GTR+I+G
	19: *nad5*_pos3	GTR+G
	20: *nad6*_pos1	GTR+G
	21: *nad6*_pos2	GTR+G

	22: *nad6*_pos3	GTR+G
	23: *cob*_pos2	GTR+I+G
	24: *cob*_pos3	GTR+G
13PCGS-amino acid - ML	1: *atp6, nad2, nad3, nad4*	mtVer+F+R5
	2: *atp8, nad5*	mtVer+F+R4
	3: *cox1*	mtVer+R4
	4: *cox2, cob*	mtVer+R4
	5: *cox3*	mtVer+R3
	6: *nad1*	mtVer+R4
	7: *nad4l*	mtVer+G4
	8: *nad6*	mtVer+F+G4
13PCGS-amino acid - BI	1: *atp6, nad1, nad2, nad4*	mtMAM+F+I+G4
	2: *atp8, nad5*	mtMAM+F+I+G4
	3: *cox1*	mtMAM+I+G4
	4: *cox2*	mtREV+F+I+G4
	5: *cox3*	mtMAM+I+G4
	6: *cob*	mtREV+I+G4
	7: *nad3*	mtREV+G4
	8: *nad6*	JTT+F+G4

Table S3: Usage of start and stop codons in the mitogenomes of three subspecies of *Platysternon*.

species	*nad1*		*nad5*		*nad2*		*cox1*		*cox2*		*atp8*		*atp6*	
	Start	Stop	Start	Stop	Start	Stop	Start	Stop	Start	Stop	Start	Stop	Start	Stop
P. m. peguense	ATG	TAG	ATG	TAA	ATG	TAG	GTG	AGG	ATG	TAG	ATG	TAA	ATG	TA(A)
P. m. megalorcephalum	ATA	TAG	ATG	TAA	ATG	TAG	ATG	AGG	ATG	TAA	ATG	TAA	ATA	TAA
P. m. shiui	ATG	TAG	ATG	TAA	ATG	TAG	ATG	AGG	ATG	TAA	ATG	TAA	ATA	TAA

species	*cox3*		*nad3*		*nad4l*		*nad4*		*nad6*		*Cob*	
	Start	Stop	Start	Stop	Start	Stop	Start	Stop	Start	Stop	Start	Stop
P. m. peguense	ATG	TA(A)	ATG	T(AA)	ATG	TAA	ATG	T(AA)	ATG	AGG	ATG	TAA
P. m. megalorcephalum	ATG	T(AA)	ATG	T(AA)	ATG	TAA	ATG	T(AA)	ATG	AGA	ATG	T(AA)
P. m. shiui	ATG	T(AA)	ATG	T(AA)	ATG	TAA	ATG	TA(A)	ATG	AGA	ATG	T(AA)

Table S4: Codon number and RSCU of *P. m. peguense* mitochondrial PCGs.

Codon	Count	RSCU	Codon	Count	RSCU	Codon	Count	RSCU	Codon	Count	RSCU
UUU(F)	105	0.92	UCU(S)	47	0.98	UAU(Y)	44	0.72	UGU(C)	13	0.79
UUC(F)	124	1.08	UCC(S)	61	1.27	UAC(Y)	79	1.28	UGC(C)	20	1.21
UUA(L)	170	1.63	UCA(S)	120	2.49	UAA(*)	8	2.46	UGA(W)	97	1.76
UUG(L)	22	0.21	UCG(S)	12	0.25	UAG(*)	3	0.92	UGG(W)	13	0.24
CUU(L)	64	0.62	CCU(P)	39	0.78	CAU(H)	37	0.67	CGU(R)	9	0.54
CUC(L)	80	0.77	CCC(P)	33	0.66	CAC(H)	73	1.33	CGC(R)	8	0.48
CUA(L)	245	2.36	CCA(P)	118	2.36	CAA(Q)	89	1.76	CGA(R)	44	2.63
CUG(L)	43	0.41	CCG(P)	10	0.2	CAG(Q)	12	0.24	CGG(R)	6	0.36
AUU(I)	159	1.07	ACU(T)	71	0.8	AAU(N)	47	0.67	AGU(S)	17	0.35
AUC(I)	137	0.93	ACC(T)	121	1.36	AAC(N)	93	1.33	AGC(S)	32	0.66
AUA(M)	180	1.54	ACA(T)	151	1.7	AAA(K)	88	1.81	AGA(*)	0	0
AUG(M)	54	0.46	ACG(T)	13	0.15	AAG(K)	9	0.19	AGG(*)	2	0.62
GUU(V)	37	0.83	GCU(A)	56	0.93	GAU(D)	20	0.66	GGU(G)	33	0.61
GUC(V)	25	0.56	GCC(A)	96	1.59	GAC(D)	41	1.34	GGC(G)	53	0.97
GUA(V)	90	2.02	GCA(A)	84	1.39	GAA(E)	73	1.64	GGA(G)	93	1.71
GUG(V)	26	0.58	GCG(A)	5	0.08	GAG(E)	16	0.36	GGG(G)	39	0.72
